# Left ventricular activation time and pattern are preserved with both selective and nonselective His bundle pacing

**DOI:** 10.1016/j.hroo.2021.08.001

**Published:** 2021-08-11

**Authors:** Ahran D. Arnold, Matthew J. Shun-Shin, Nadine Ali, Daniel Keene, James P. Howard, Ji-Jian Chow, Norman A. Qureshi, Michael Koa-Wing, Mark Tanner, David C. Lefroy, Nick W.F. Linton, Fu Siong Ng, Phang Boon Lim, Nicholas S. Peters, Prapa Kanagaratnam, Darrel P. Francis, Zachary I. Whinnett

**Affiliations:** National Heart and Lung Institute, Imperial College London, London, United Kingdom

**Keywords:** Pacing, ECG, His bundle pacing, Electrocardiographic imaging, Selective His bundle pacing, Conduction system pacing

## Abstract

**Background:**

His bundle pacing (HBP) can be achieved in 2 ways: selective HBP (S-HBP), where the His bundle is captured alone, and nonselective HBP (NS-HBP), where local myocardium is also captured, resulting a pre-excited electrocardiogram appearance.

**Objective:**

We assessed the impact of this ventricular pre-excitation on left and right ventricular dyssynchrony.

**Methods:**

We recruited patients who displayed both S-HBP and NS-HBP. We performed noninvasive epicardial electrical mapping for left and right ventricular activation time (LVAT and RVAT) and pattern.

**Results:**

Twenty patients were recruited. In the primary analysis, the mean within-patient change in LVAT from S-HBP to NS-HBP was -5.5 ms (95% confidence interval: -0.6 to -10.4, noninferiority *P* < .0001). NS-HBP did not prolong RVAT (4.3 ms, -4.0 to 12.8, *P* = .296) but did prolong QRS duration (QRSd, 22.1 ms, 11.8 to 32.4, *P* = .0003). In patients with narrow intrinsic QRS (n = 6), NS-HBP preserved LVAT (-2.9 ms, -9.7 to 4.0, *P* = .331) but prolonged QRS duration (31.4 ms, 22.0 to 40.7, *P* = .0003) and mean RVAT (16.8 ms, -5.3 to 38.9, *P* = .108) compared to S-HBP. Activation pattern of the left ventricular surface was unchanged between S-HBP and NS-HBP, but NS-HBP produced early basal right ventricular activation that was not seen in S-HBP.

**Conclusion:**

Compared to S-HBP, local myocardial capture during NS-HBP produces pre-excitation of the basal right ventricle resulting in QRS duration prolongation. However, NS-HBP preserves the left ventricular activation time and pattern of S-HBP. Left ventricular dyssynchrony is not an important factor when choosing between S-HBP and NS-HBP in most patients.

## Graphical abstract

**Figure:**
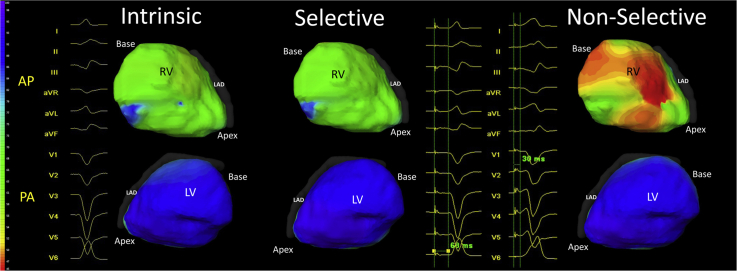

**Key Findings**

▪ Nonselective His bundle pacing preserves the left ventricular activation time and pattern of selective His bundle pacing
▪ Nonselective His bundle pacing prolongs QRS duration compared to selective His bundle pacing.
▪ The QRS duration prolongation from nonselective His bundle pacing appears to be due to right ventricular activation time prolongation.
▪ Right ventricular activation time prolongation with nonselective His bundle pacing is due to basal right ventricular pre-excitation.
▪ In right bundle branch block, nonselective His bundle pacing can shorten right ventricular activation time.

## Introduction

His bundle pacing (HBP) has emerged as a paradigm shift in pacemaker therapy. Conventional ventricular pacing results in slow cell-cell myocardial conduction, resulting in a broad QRS complex.^1^ By activating the ventricles via the His-Purkinje conduction system, HBP produces a narrower QRS than right ventricular pacing (RVP) in patients whose unpaced QRS appearance is normal. However, HBP does not always produce an identical QRS to the intrinsic, unpaced QRS. This only occurs in *selective* His bundle pacing (S-HBP) where the His bundle alone is captured by the pacing stimulus. S-HBP results in an electrocardiographic appearance of an isoelectric interval between the pacing stimulus and QRS onset (Stim-V interval) of similar duration to the intrinsic HV interval. In *nonselective* His bundle pacing (NS-HBP) the local myocardium surrounding the His bundle is captured alongside the His bundle itself. QRS prolongation therefore occurs owing to slow cell-to-cell myocardial conduction during the initial poststimulus period, where there would have been an isoelectric interval in S-HBP.

NS-HBP has the potential advantage of providing continuous pacing even if conduction system block develops distal to the pacing site. However, it is not known if the QRS widening seen in NS-HBP occurs only owing to isolated prolongation of right ventricular activation or if it also represents dyssynchronous left ventricular activation, which could have detrimental effects on cardiac function. This has important implications for implantation and programming for HBP.

In this study, we used high-resolution noninvasive epicardial mapping to measure the within-patient effect of HBP selectivity on intraventricular electrical synchrony.

## Methods

### Study population

Patients undergoing HBP were recruited if both S-HBP and NS-HBP were observed in the same individual patient. S-HBP and NS-HBP were defined according to internationally recognized criteria (set out in the Online Appendix).^1^, ^2^, ^3^ Patients with various indications for HBP were recruited: resynchronization of bundle branch block (BBB), optimization of atrioventricular interval in heart failure, or prevention of pacing-induced cardiomyopathy in atrioventricular block. All patients gave written, informed consent and the study was approved by the local ethics committee (13/LO/1440). The research reported in this paper adhered to the Helsinki Declaration.

### Noninvasive epicardial electrical mapping

All patients were fitted with a 252-electrode noninvasive epicardial electrical mapping (ECGI) vest (Medtronic, Minneapolis, MN) and underwent low-dose thoracic computed tomography for cardiac anatomy and electrode positions. The ECGI methodology has been described and validated in previous work: multielectrode body-surface potentials are combined with radiologically acquired epicardial anatomy using the ECGI solution to the inverse problem to reconstruct unipolar epicardial electrograms. ECGI recordings were made during intrinsic rhythm, S-HBP, and NS-HBP.

### Activation time analysis

Activations from individual electrodes were temporally annotated based on the most negative dv/dt (the steepest slope of the voltage-time relationship) and visualized on patients’ 3-dimensional cardiac model. The total left ventricular activation time (LVAT) was calculated from earliest to latest activation. This value can be skewed by outliers caused by noise and anatomical and temporal mis-annotation; therefore the activation time of 95% of activations (LVAT_95_) was used to quantify resynchronization.^4^ Right ventricular activation time (RVAT_95_) was similarly calculated.

### Activation pattern analysis

Activation patterns were analyzed using both ECGI activation maps and ECGI-derived epicardial propagation maps. To create epicardial propagation map cines, custom software was used to visualize wavefront propagation across the epicardium. Enlarging circles, with the circle radius proportionate to the rectified epicardial potential amplitude as it varied over the cardiac cycle, were displayed for each virtual epicardial electrode on a 3-dimensional cardiac model. By displaying the entire electrogram for each electrode, rather than the most negative dv/dt, visual interpretation was used to determine activation wavefronts rather than relying on potentially mis-annotated activations.

### Pacing

There were 2 groups of patients recruited. The first group comprised patients undergoing temporary HBP as part of a research protocol performed during conventional biventricular pacing (BVP) implants. These patients had intraprocedural ECGI measurements, and HBP was performed for attempted correction of left bundle branch block (LBBB). These patients were included only when both selective and nonselective HBP both produced correction of LBBB or both failed to produce correction of LBBB. If the femoral route was used, a quadripolar electrophysiology catheter was placed on the bundle of His. If the subclavian route was used, a SelectSecure 3830 lead was delivered via either a C304-His deflectable sheath or C315 fixed curve sheath (leads and delivery system: Medtronic). The lead was not actively fixated unless BVP failed, in which case the SelectSecure 3830 lead was deployed for permanent HBP.

The second group of patients had permanent SelectSecure 3830 HBP leads implanted prior to recruitment, for clinical indications. ECGI measurements in these patients were performed at least 6 weeks after implantation.

### Statistical analysis

Greater than 10 ms reduction in LVAT_95_ by HBP has previously been defined as resynchronization^4^: Changes in LVAT_95_ that are <10 ms cannot be distinguished from measurement variation. Therefore, the primary analysis was powered with a noninferiority margin of 10 ms LVAT_95_ prolongation by NS-HBP compared to S-HBP. From previous analysis of HBP LVAT_95_,^4^ we determined the reproducibility standard deviation of HBP to be 7 ms. To demonstrate that NS-HBP LVAT_95_ is statistically noninferior (not prolonged by >10 ms) to S-HBP, 18 patients provide 80% power at the 0.05 significance level. Planned secondary analyses are set out in the Online Appendix. Paired *t* tests were used for within-patient comparisons. Statistical analyses were performed using the statistical environment “R” with the “ggplot2” visualization package (R Foundation for Statistical Computing, Vienna, Austria).

## Results

**Figure 1:**
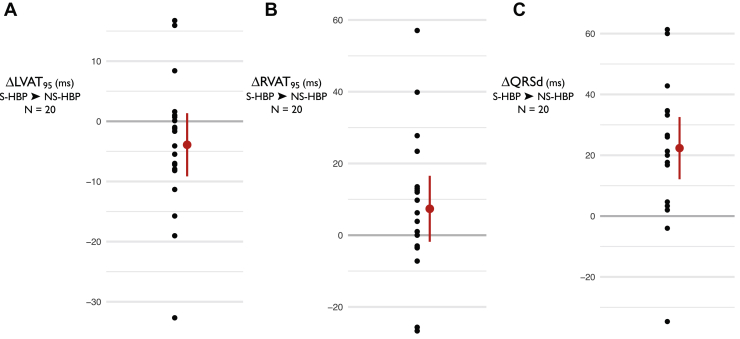
Change in activation times from selective His bundle pacing (S-HBP) to nonselective His bundle pacing (NS-HBP) for LVAT_95_ (**A**), RVAT_95_ (**B**), and QRSd (**C**) is shown for all patients (n = 20). LVAT_95_ = left ventricular activation time of 95% of activations; QRSd = QRS duration; RVAT_95_ = right ventricular activation time of 95% of activations.

**Figure 2:**
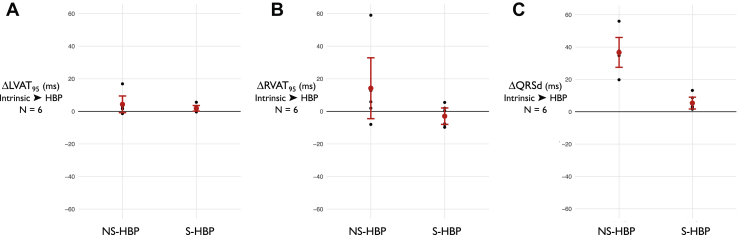
Change in activation times from intrinsic to His bundle pacing (HBP) (patients with narrow intrinsic QRS). The change from intrinsic to selective HBP (S-HBP) and from intrinsic to nonselective HBP (NS-HBP) for LVAT_95_ (**A**), RVAT_95_ (**B**), QRSd (**C**) is shown for patients with a narrow intrinsic QRS (n = 6). LVAT_95_ = left ventricular activation time of 95% of activations; QRSd = QRS duration; RVAT_95_ = right ventricular activation time of 95% of activations.

Twenty subjects were recruited; their baseline demographics are displayed in Table 1. The majority of patients had intrinsic BBB and the most common indication was resynchronization of LBBB. Activation time and QRS duration (QRSd) results are set out in Table 2 and Figures 1 and 2 and all activation parameters are displayed for reference in the Online Appendix (Supplemental Tables S1 and S2, Supplemental Figures S1 and S2).

**Table 1:** Baseline characteristics

Parameter	Value
Age	69.7 ± 12.3 (46–88)
Male	16 (80%)
PR interval	225.7 ± 11.6 (130–396)
HV interval†	49.6 ± 20.8 (26–106)‡
Narrow QRS	6 (30%)
LBBB	12 (60%)
RBBB	2 (10%)
Ejection fraction	31.3 ± 11.6 (14–67.3)
Ischemic heart disease	11 (55%)
NYHA grade	2.4 ± 0.7 (2–4)
Beta blockers	18 (90%)
ACE inhibitors	19 (95%)
MRA	12 (60%)
Sacubitril	2 (10%)

Values are mean ± SD (range) or n (%).

ACE = angiotensin-converting enzyme; ARB = angiotensin receptor blocker; LBBB = left bundle branch block; RBBB = right bundle branch block.

† HV interval is time from His signal to onset of QRS in intrinsic rhythm.

‡ The values for patients with narrow QRS were mean 35.7 ± 4.6, range 30–42.

**Table 2:** Results

Parameter	LVAT_95_, ms	RVAT_95_, ms	QRSd, ms
All patients (n = 20)			
Δ S-HBP → NS-HBP	-5.5 (-0.6 to -10.4) *P* = .030∗	4.3 (-4.0 to 12.8) *P* = .296	22.1 (11.8 to 32.4) *P* = .0003
Patients with narrow QRS (n = 6)			
Δ S-HBP → NS-HBP	-2.9 (-9.7 to 4.0) *P* = .331	+16.8 (-5.3 to 38.9) *P* = .108	+31.4 (22.0 to 40.7) *P* = .0003
Δ Intrinsic → S-HBP	+4.5 (-2.2 to 11.3) *P* = .143	-2.9 (-9.6 to 3.7) *P* = .306	+5.4 (0.6 to 10.2) *P* = .034

Values are mean, 95% CI, *P* value. *P* values are for superiority 2-tailed paired *t* tests. Δ S-HBP → NS-HBP is the within-patient change in parameter value from S-HBP to NS-HBP. Δ Intrinsic → S-HBP is the within-patient change in parameter value from intrinsic to S-HBP.

LVAT_95_ = left ventricular activation time of 95% of activations; NS-HBP = nonselective His bundle pacing; QRSd = QRS duration; RVAT_95_ = right ventricular activation time of 95% of activations; S-HBP = selective His bundle pacing.

∗ This is the superiority *P* value; noninferiority *P* value is in main text.

### Activation times and QRS durations

In the primary analysis, the mean within-patient change in LVAT_95_ from S-HBP to NS-HBP for all 20 patients was -5.5 ms (ie, LVAT_95_ was 5.5 ms shorter with NS-HBP), 95% confidence interval: -0.6 to -10.4 ms, n = 20. NS-HBP LVAT_95_ was statistically noninferior to S-HBP (noninferiority *P* < .0001). RVAT_95_ was also not prolonged by NS-HBP compared to S-HBP but QRSd was on average 22.1 ms longer with NS-HBP compared to S-HBP (11.8 to 32.4 ms, *P* = .0003).

**Figure 3:**
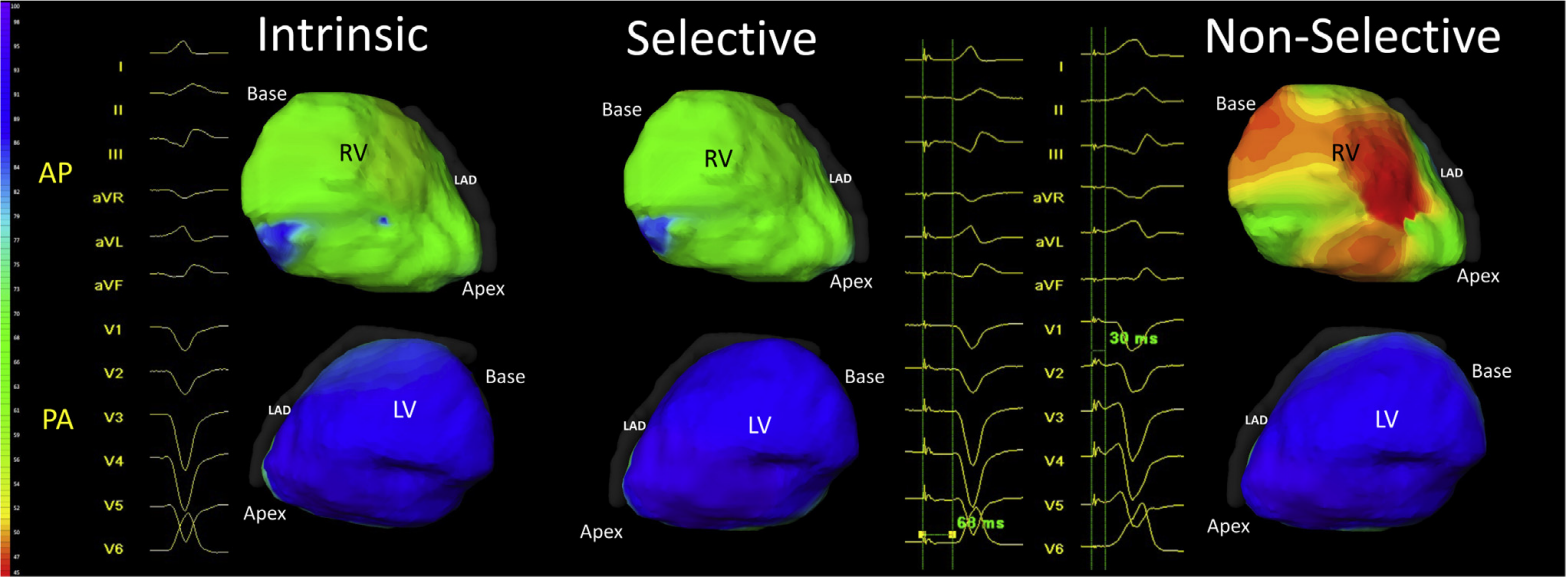
Left ventricular activation pattern is preserved by nonselective His bundle pacing (NS-HBP). ECGI epicardial activation maps (Medtronic, Minneapolis, MN) of intrinsic (left), selective His bundle pacing (S-HBP) (middle), and NS-HBP (right) of the right ventricle (RV; top) and left ventricle (LV; bottom) with 12-lead electrocardiograms (ECGs), all from a single patient. The color scale on the left demonstrates the timing of activation. The 12-lead ECG of S-HBP demonstrates a physiological isoelectric interval between stimulation artefact and QRS onset, followed by a QRS complex identical to intrinsic QRS in morphology and duration indicating selective capture of the His bundle has been achieved, without local myocardial capture. LV and RV activation patterns are preserved from intrinsic to S-HBP. In NS-HBP, QRS onset occurs very soon after the stimulation artefact and there is a slurred onset of QRS before the remainder of the QRS becomes similar in morphology to the latter part of the QRS in S-HBP and intrinsic rhythm. LV activation appears similar to S-HBP and intrinsic rhythm. On the epicardial surface of the RV, however, early (*red*) activation can be seen in the basal to mid region, which is not seen in S-HBP or intrinsic rhythm. This suggests that NS-HBP pre-excites the RV (producing some RV dyssynchrony) but preserves LV activation.

In patients with a narrow intrinsic QRS complex (n = 6), LVAT_95_ was not prolonged by NS-HBP compared to S-HBP. NS-HBP produced a mean 16.8 ms increase in RVAT_95_ compared to S-HBP; this finding did not meet statistical significance (-5.3 to 38.9 ms, *P* = .108). QRSd was significantly prolonged by NS-HBP by an average of 31.4 ms compared to S-HBP in these patients (22.0 to 40.7, *P* = .0003). Example electrocardiograms of S-HBP and NS-HBP are shown in Figure 3 and Supplemental Figure S3.

### Activation maps

ECGI activation maps of S-HBP and NS-HBP demonstrated that left ventricular activation pattern appears unchanged from intrinsic rhythm to S-HBP and to NS-HBP. Right ventricular activation appears unchanged from intrinsic rhythm to S-HBP. NS-HBP, however, displays early activation in the basal right ventricle (RV), consistent with capture of local myocardium alongside the His bundle. Example ECGI activation maps from a patient with narrow intrinsic QRS are shown in Figure 3.

### Propagation maps

Example ECGI epicardial propagation cines from the same patient can be viewed in the accompanying video in the Online Appendix. The left ventricle (LV) is activated smoothly and rapidly and the pattern is unchanged from intrinsic rhythm during both S-HBP and NS-HBP. The RV is also activated smoothly and rapidly in intrinsic rhythm and S-HBP. In NS-HBP a slowly conducting wavefront travels from the basal to mid RV before colliding with a rapidly conducting wavefront that activates the remaining RV surface, while LV activation is unchanged.

## Discussion

In this study we measured the effects of HBP selectivity on left and right ventricular activation time and pattern using high-resolution epicardial mapping. There were several important findings: (1) NS-HBP preserves left ventricular electrical synchrony, despite prolonging QRS duration, when compared to S-HBP; (2) NS-HBP produces some prolongation of right ventricular activation time compared to S-HBP, which contributes to QRSd prolongation; (3) NS-HBP produces pre-excitation of the basal right ventricular epicardium, contributing to QRSd prolongation, but does not alter left ventricular activation pattern compared to S-HBP.

### QRS prolongation

In patients with normal, narrow baseline QRS, NS-HBP necessarily entails a degree of QRSd prolongation compared to intrinsic QRS; indeed, it is defined by it: shortening of Stim-V with preservation of Stim-QRS_end_ are criteria for NS-HBP and must result in a longer QRS complex than with S-HBP. The “underlying” QRSd of NS-HBP can be calculated as Stim-QRS_end_ minus HV interval or estimated as the time to QRS offset from the inflection at the junction between the slurred pseudo-delta-wave and the sharp deflection of a normal QRS. However, to exclude NS-HBP QRS prolongation in this way is to assume it has no importance. Large observation analyses suggest that QRS prolongation (whether through RVP or BBB) is associated with worsened ventricular function and higher mortality.^5^ There is randomized controlled trial evidence of this phenomenon: In patients with heart failure and narrow QRS, BVP (which prolongs QRSd in this context) worsens mortality.^6^ In these patients LVAT is prolonged.^7^

### HBP selectivity and left ventricular synchrony

NS-HBP, however, has not been associated with worsened outcomes when compared to S-HBP in observational analysis,^8^ despite QRS prolongation. The prevailing model of HBP as physiological pacing and LV intraventricular dyssynchrony as a driver for ventricular impairment in QRS prolongation support the disparate impact of QRSd prolongation in NS-HBP compared to other causes of wide QRS (RVP, BVP, BBB). HBP is invariably performed from the right side and the His bundle itself has been histologically^9^ located in the right side of the septum. Therefore, it has been speculated that in NS-HBP the tissue activated local to the *right-sided* His bundle and the *right-sided* His lead electrode would be *right* ventricular myocardium.^10^ Thus, RV activation would be mainly affected by NS-HBP, preserving LV synchrony. Whereas in RVP, BVP, and BBB (other causes of wide QRS), dyssynchronous *left* ventricular activation is expected and has been observed.^11^ Our previous work has shown a direct relationship between more synchronous LV activation time, assessed using the same ECGI technique in this study, and improved hemodynamics, suggesting that intra-LV electrical synchrony is a key driver of cardiac output.^4^ Our findings are consistent with this model, as LV synchrony was preserved by NS-HBP.

### HBP selectivity and right ventricular synchrony

The point estimate for RV electrical dyssynchrony (RVAT_95_) was increased by NS-HBP in patients with intrinsically narrow QRS complexes. Although this did not reach statistical significance, the sample size for this subgroup analysis (n = 6) was not, a priori, powered for. The mean within-patient increase in RVAT_95_ was 16.8 ms. Although this is modest compared to HBP resynchronization induced changes in activation time, which can be several times larger,^4^ it is not necessarily trivial. BVP produces a similar magnitude of LVAT_95_ reduction in LBBB and this has important clinical effects of reduced mortality and morbidity.^12^ Although NS-HBP did not prolong RVAT_95_ in the group of all patients, this group includes patients with right bundle branch block (RBBB), in whom NS-HBP is known to produce RV resynchronization,^13^ and patients with LBBB where RV dyssynchrony can be masked by the broad LBBB QRS. Therefore, assessment of NS-HBP-induced RVAT_95_ dyssynchrony may be confounded by RV resynchronization in these patients. Indeed, there was substantial shortening of RVAT_95_ in the 2 patients with RBBB (>50 ms) and some shortening in patients with LBBB (>20 ms). An assumption of the model of clinically beneficial preservation of LV synchrony by NS-HBP is that RV dyssynchrony is less important than LV dyssynchrony. This is borne out in observational analysis of patients with heart failure and LBBB suffering worse outcomes than those with RBBB^14^ in the pre-cardiac resynchronization therapy era. There is some evidence that RV dyssynchrony can be clinically important,^15^ but the magnitude of RVAT prolongation we observed with NS-HBP was small.

### Selective HBP vs intrinsic narrow QRS

Importantly, our results are consistent with the fundamental promise of HBP, which is that by activating the ventricles physiologically via the His-Purkinje system, HBP can preserve ventricular synchrony. In this study we demonstrated this using epicardial mapping: S-HBP did not prolong left or right ventricular activation time compared to intrinsic QRS. This helps to explain the observed association between HBP and successful prevention and treatment of pacing-induced cardiomyopathy.^16^^,^^17^

### Activation patterns

In patients with intrinsically narrow QRS and patients with uncorrected LBBB, the left ventricular activation pattern was indistinguishable between S-HBP, NS-HBP, and intrinsic activation. In patients with narrow QRS, this was smooth, rapid activation of the left ventricular epicardial surface. Mean LVAT_95_ for these patients was within the normal range of patients with normal QRS and normal LV function.^18^ RV maps demonstrated early basal-mid activation consistent with capture of right ventricular myocardium from a position close to the lead tip.

### Clinical differences between S-HBP and NS-HBP

A concern for the use of HBP in patients with atrioventricular block indications is the risk of progression of conduction system disease and the development of infra-Hisian block (Supplemental Figure S3). In this context there is a risk of loss of ventricular capture with S-HBP. While this phenomenon has not been observed in observational studies performed with HBP to date, the potential risk has led operators to implant a back-up right ventricular lead (or avoid HBP). NS-HBP would allow ventricular capture to be preserved in the event of a patient’s developing infra-Hisian block. When this block occurs, NS-HBP will (functionally) become myocardium-only capture, producing a high RV septal paced QRS complex. Although this produces dyssynchronous activation, which is not aimed for in HBP, it allows continued pacing. NS-HBP may therefore be preferable in patients at risk of developing infra-Hisian block. However, what has not previously been known is whether pursuing NS-HBP will attenuate the ability of HBP to deliver physiological, synchronous left ventricular activation. This has resulted in a difficult judgment for physicians managing patients with His leads.

Our findings suggest that in most patients NS-HBP does not affect LV synchrony and therefore that this is not an important factor in capture type selection. Our findings are consistent with other clinical and synchrony assessments of HBP selectivity. Curila and colleagues^19^ found LV electrical synchrony unchanged between S-HBP and NS-HBP; Zhang and colleagues^20^ found the same for LV mechanical synchrony. Beer and colleagues’ observational study^8^ found no statistically significant difference in death or heart failure hospitalization between S-HBP and NS-HBP, even when analyzing high-risk subgroups. Our findings add to these by allowing precise quantification of activation times, right ventricular synchrony assessment, and activation pattern detail, which can only be analyzed using high-resolution electrical mapping such as the method used in this study. Therefore the totality of evidence, including our findings, suggests that NS-HBP can be safely targeted in most patients. It should be noted, however, that there may be reasons, other than dyssynchrony, to favor S-HBP or NS-HBP, including the stability of thresholds, for example.

### NS-HBP-induced dyssynchrony

We do not infer from our results that NS-HBP preserves LVAT_95_ in all patients: This was the mean effect. In 1 patient, LVAT_95_ was prolonged by greater than the 10 ms noninferiority margin (16.8 ms). Some outliers are expected owing to natural biological and measurement variation, but there is also likely to be a subset of patients with long intrinsic HV intervals in whom left ventricular activation is prolonged by NS-HBP. HV intervals in patients with conduction disease can be very long, including >100 ms (if this is not corrected by HBP). This allows a long period of time for even slow cell-to-cell myocardial propagation to activate a large region of right ventricular myocardium and perhaps break through the septum to activate a large amount of left ventricular myocardium before His-Purkinje activation overtakes. This could be enough to produce important LV dyssynchrony: Patients with accessory pathway–induced pre-excitation, in rare cases, can have dyssynchrony-induced symptoms that resolve with ablation.^21^ In patients with normal or only modestly prolonged HV intervals, there is likely to be a small amount of left ventricular pre-excitation confined to the basal septum, which is not seen on epicardial ECGI analysis.

### Limitations

The sample size was prospectively powered for the primary analysis, but subgroups were small. The RVAT_95_ prolongation seen with NS-HBP with patients with narrow intrinsic QRS complex, for example, may have been statistically significant with a larger sample size. The ECGI methodology has been validated previously but makes several assumptions, including static geometry, and does not image the septum,^22^ but the bulk of myocardium is assessed and LVAT_95_ changes have been found to correlate with changes in cardiac output. ECGI is less well studied for assessment of RV dyssynchrony. The assessment of LV synchrony was acute but was consistent with the long-term clinical outcomes observed by Beer and colleagues.^8^ Only 3 patients had ejection fractions of >35% and just 1 of these patients had normal LV function. It may not be possible to fully extrapolate these findings to patients with normal LV function. Patients with narrow intrinsic QRS had SelectSecure 3830 leads deployed into the His bundle paced in bipolar configuration, whereas patients studied intraprocedurally had electrophysiological catheters or undeployed SelectSecure leads paced in the unipolar configuration. This has the potential to affect the virtual electrode produced, and thereby affect the degree of nonselectivity of capture.

## Conclusion

NS-HBP preserves left ventricular electrical synchrony, despite prolonging QRS duration, when compared to S-HBP. NS-HBP can be safely targeted in most patients.

## Funding Sources

This study was funded by the 10.13039/501100000274British Heart Foundation (FS/13/44/30291, FS/CRTF/21/24171). Dr Arnold is supported the 10.13039/501100005617BHF Imperial Centre of Research Excellence (RE/18/4/34215) and the NIHR Imperial Biomedical Research Centre.

## Disclosures

The authors have no conflicts to disclose.

## Authorship

All authors attest they meet the current ICMJE criteria for authorship.

## Patient Consent

All patients gave written, informed consent.

## Ethics Statement

The study was approved by the local ethics committee (13/LO/1440). The research reported in this paper adhered to the Helsinki Declaration.
