# Supplementary material for: Left ventricular activation time and pattern are preserved with both selective and nonselective His bundle pacing
Source: Heart Rhythm O2. 2021 Aug 11;2(5):439–45. doi: 10.1016/j.hroo.2021.08.001 (PMC8505200; doi:10.1016/j.hroo.2021.08.001)
Supplement: Supplementary Material [file mmc1.docx]

Online Appendix

# **Online Video**

The online video shows epicardial propagation mapping cines in the following order:

1. **Selective His bundle pacing: view of left ventricle**

This is a propagation of map of selective His bundle pacing with left bundle branch block that is uncorrected by His bundle pacing at this pacing output. On the left ventricular epicardial surface there is an appearance of discontinuity with diffusely slow ventricular activation originating from the posterior aspect of the left ventricle.

1. **Non-selective His bundle pacing: view of left ventricle**

The pacing output is raised to achieve non-selective His bundle pacing but left bundle branch block remains uncorrected. The left ventricular activation pattern and time is unchanged from selective His bundle pacing.

1. **Selective His bundle pacing: view of right ventricle**

The right ventricle is activated rapidly and smoothly in selective His bundle pacing.

1. **Non-selective His bundle pacing: view of left ventricle**

In non-selective His bundle pacing, a small region of the basal right ventricle is activated much earlier (bright red) and soon after this the remainder of the right ventricle is activated smoothly and rapidly.

# **Extended Methods**

## Definitions Of Capture

Selective and non-selective His bundle capture were determined using standard criteria as follows. S-HBP was defined as a stimulation to QRS offset during pacing (Stim-QRS_end_) equal to the interval in intrinsic rhythm from His signal to QRS offset (H-QRS_end_) with Stim-V interval equal to HV interval. NS-HBP was defined as a Stim-QRS_end_ interval equal to or lower than the H-QRS_end_ interval with Stim-V interval both less than 30ms and less than the HV interval. In the case of bundle branch block (BBB) correction, a shortening of Stim- QRS_end_ from H-QRS_end_ is expected. QRS duration was measured from observable QRS onset to QRS offset and therefore may provide some insight into septal activation in NS-HBP.

If Stim-QRS_end_ was longer than H-QRS_end_, this was excluded as myocardial only capture. Myocardial-only capture (also referred to as septal pacing) can produce an equal or slightly shorter Stim-QRS_end_ than H-QRS_end_ in BBB, which can appear similar to NS-HBP with partial BBB correction. In such cases NS-HBP was discriminated from myocardial capture by change in QRS morphology and/or Stim- QRS_end_ interval when increasing pacing output, which confirms conduction system capture. Although myocardial-only capture and S-HBP are not observed in the same patient via the same permanently implanted His lead, multiple HBP attempts can produce the two responses in different attempts.

## Planned analyses

The primary planned analysis is set out in the main text. Planned secondary analyses were as follows: 1) comparing RVAT-95 between intrinsic rhythm, S-HBP and NS-HBP, 2) comparing LVAT-95 between intrinsic rhythm and S-HBP in patients with where bundle branch block (BBB) was either not present at baseline or not corrected by HBP, 3) comparing RVAT-95 between S-HBP and NS-HBP in patients with left bundle branch block (LBBB) and right bundle branch block (RBBB). LVAT_95_ was not compared between intrinsic rhythm and HBP in patients where LBBB was corrected by HBP as the purpose of HBP in this circumstance is to shorten LVAT_95_ by recruiting the left bundle.

**3. Extended Results and Discussion: Sub-Group Analyses**

On the following pages:

**Supplementary Table S1: Activation times for all sub-groups**

**Sub-group Analysis Results**

**Supplementary Table S2: Results narrow QRS and uncorrected LBBB**

**Sub-group Analysis Discussion**

| **Parameter** | **LVAT_95_, ms** | **RVAT_95_, ms** | **QRSd, ms** |
| --- | --- | --- | --- |
| **All patients (n = 20)** | | | |
| **Intrinsic** | 90.6 ± 30.5  (46.9 – 143.1) | 63.3 ± 30.2  (17.6 – 118.4) | 148.2 ± 33.4  (86.0 – 192.7) |
| **Selective HBP** | 87.2 ± 22.9  (48.3 – 129.3) | 52.6 ± 24.7  (7.8 – 104.3) | 141.8 ± 29.9  (87.6 – 191) |
| **Non-Selective HBP** | 81.7 ± 20.3  (49.6 – 131.8) | 57.0 ± 20.8  (31.0 – 117.3) | 163.9 ± 26.5  (121 – 224) |
| **∆ S-HBP → NS-HBP** | -5.5 ± 10.4  (-32.7 – 16.8) | 4.3 ± 4.0  (-26.7 – 54.8) | 22.1 ± 22.0  (-34.7 – 61.3) |
| **∆ Intrinsic → S-HBP** | -3.4 ± 20.1  (-47.9 – 36.0) | -10.7 ± 21.9  (-74.8 – 14.0) | -6.4 ± 17.0  (-42 – 13.2) |
| **Patients with Narrow QRS (n = 6)** | | | |
| **Intrinsic** | 61.0 ± 11.1  (46.8 – 73.4) | 30.4 ± 12.8  (17.6 – 44.4) | 102.1 ± 14.8  (86 – 119) |
| **Selective HBP** | 65.6 ± 15.4  (48.3 – 90.3) | 27.5 ± 14.5  (7.8 – 49.9) | 107.4 ± 13.1  (87.6 – 122) |
| **Non-Selective HBP** | 62.7 ± 10.7  (49.6 – 74.4) | 44.3 ± 16.9  (31 – 75.0) | 138.7 ± 11.7  (121 – 156) |
| **∆ S-HBP → NS-HBP** | -2.9 ± 6.5  (-15.9 – 1.6) | 16.8 ± 21.0  (-3.5 – 54.8) | 31.4 ± 8.9  (16.8 – 42.8) |
| **∆ Intrinsic → S-HBP** | 4.5 ± 6.4  (-0.3 – 16.9) | -2.9 ± 6.3  (-9.8 – 5.5) | 5.4 ± 4.6  (1.6 – 13.2) |
| **Patients with narrow QRS or uncorrected LBBB (n = 15)** | | | |
| **Intrinsic** | 84.8 ± 28.1  (46.8 – 129.0) | 51.4 ± 24.1  (17.6 – 97.0) | 142.3 ± 36.2  (86 – 192.7) |
| **Selective HBP** | 89.1 ± 22.6  (48.3 – 129.3) | 45.5 ± 20.1  (7.8 – 74.4) | 141.0 ± 32.3  (87.6 – 191) |
| **Non-Selective HBP** | 82.9 ± 22.6  (49.6 – 131.8) | 53.0 ± 15.6  (31.0 – 75.0) | 159.3 ± 27.0  (121 – 224) |
| **∆ S-HBP → NS-HBP** | -6.2 ± 11.2  (-32.7 – 16.8) | 7.4 ± 18.5  (-26.7 – 54.8) | 18.3 ± 22.8  (-34.7 – 60.0) |
| **∆ Intrinsic → S-HBP** | 4.3 ± 11.8  (-10.0 – 36.0) | -5.9 ± 12.5  (-32 – 10.7) | -1.2 ± 13.1  (-42 – 13.2) |
| **Patients with resynchronized LBBB (n = 3)** | | | |
| **Intrinsic** | 132.2 ± 9.5  (125.8 – 143.1) | 93.6 ± 8.2  (87.5 – 102.9) | 172.3 ± 21.2  (133.3 – 170) |
| **Selective HBP** | 89.1 ± 9.6  (125.8 – 143.1) | 77.2 ± 27.4  (49.5 – 104.3) | 157.8 ± 21.2  (133.3 – 170) |
| **Non-Selective HBP** | 81.7 ± 12.9  (71.0 – 96.0) | 76.6 ± 39.9 (37.5 – 117.3) | 193.6 ± 3.2  (190 – 196) |
| **∆ S-HBP → NS-HBP** | -7.3 ± 8.2  (-15.7 – 0.7) | -0.7 ± 12.6  (-12 – 13.0) | 35.8 ± 22.3  (20 – 61.3) |
| **∆ Intrinsic → S-HBP** | -43.1 ± 8.2  (-47.8 – -33.6) | -16.3 ± 34.2  (-53.4 – 14.0) | -14.6 ± 23.3  (-35.3 – 10.7) |
| **Patients with resynchronised RBBB (n = 2)** | | | |
| **Intrinsic** | 71.5 ± 8  (65.7 – 77.3) | 107.7 ± 15.1  (97.0 – 118.4) | 156.4 ± 1.3  (155.4 – 157.3) |
| **Selective HBP** | 69.9 ± 4.1  (67 – 72.8) | 68.8 ± 35.6  (97 – 118.4) | 123.6 ± 3.16  (121.3 – 125.8) |
| **Non-Selective HBP** | 72.1 ± 12.9  (62.9 – 81.2) | 57.5 ± 15.3  (46.6 – 68.3) | 154.2 ± 2.5  (152.4 – 156.0) |
| **∆ S-HBP → NS-HBP** | 2.2 ± 8.8  (-4.1 – 8.4) | -11.3 ± 20.3  (-25.7 – 2.98) | 30.6 ± 5.7  (26.6 – 34.7) |
| **∆ Intrinsic → S-HBP** | -1.6 ± 4.1  (-4.5 – 1.3) | -38.9 ± 50.8  (-74.8 – -3) | -32.8 ± 4.5  (-36.0 – -29.6) |

**Supplementary Table S1: Activation Times**

Values are mean ± SD (range)

∆ S-HBP → NS-HBP is the change in parameter value from S-HBP to NS-HBP

∆ Intrinsic → S-HBP is the change in parameter value from Intrinsic to S-HBP

LVAT_95_ – left ventricular activation time of 95% of activations; RVAT_95_ – right ventricular activation time of 95% of activations; QRSd – QRS duration; S-HBP – selective His bundle pacing; NS-HBP – non-selective His bundle pacing.

Sub-Group Results
In patients with either a narrow intrinsic QRS or uncorrected bundle branch block (n=15), S-HBP did not affect LVAT_95_ or RVAT_95_ compared to intrinsic unpaced QRS or NS-HBP. QRSd was prolonged on average by 18.3 ms by NS-HBP compared to S-HBP (73 to 19.2 ms, p=0.008) but QRSd was not prolonged compared to intrinsic unpaced QRS, in these patients.

| **Parameter** | **LVAT_95_, ms** | **RVAT_95_, ms** | **QRSd, ms** |
| --- | --- | --- | --- |
| **Patients with narrow QRS or uncorrected LBBB (n = 15)** | | | |
| **∆ S-HBP → NS-HBP** | -6.2  (-11.5 to -0.8)  *p = 0.051* | 7.4  (-1.4 to 16.3)  p = 0.141 | 18.3  (7.3 to 29.2)  **p = 0.008** |
| **∆ Intrinsic → S-HBP** | 4.3  (-1.4 to 9.9)  p = 0.183 | -5.9  (-11.9 to 0.1)  *p = 0.089* | -1.2  (-7.5 to 5.1)  p = 0.724 |

**Supplementary Table S2: Results for patients with narrow QRS or uncorrected LBBB**

Values are mean, 95% Confidence Interval, P value. P values are for superiority two-tailed paired t tests. ∆ S-HBP → NS-HBP is the within-patient change in parameter value from S-HBP to NS-HBP. ∆ Intrinsic → S-HBP is the within-patient change in parameter value from Intrinsic to S-HBP.

LVAT_95_ – left ventricular activation time of 95% of activations; RVAT_95_ – right ventricular activation time of 95% of activations; QRSd – QRS duration; S-HBP – selective His bundle pacing; NS-HBP – non-selective His bundle pacing; LBBB – left bundle branch block.

## HBP for resynchronization of RBBB

Both S-HBP and NS-HBP can resynchronize RBBB but through different mechanisms. S-HBP can recruit the right bundle distal to the region of block. NS-HBP produces more than one wavefront in the RV: one is basal, from the His bundle lead, and others more apical, the regions where left bundle mediated activation breaks out into the RV. The effect of multiple wavefronts is to more rapidly complete activation of the RV, even though the right bundle is not necessarily recruited. These mechanisms were observed in our dataset. In one patient, RBBB prolongation of RVAT_95_ was reduced by both S-HBP and NS-HBP (indicating bundle recruitment). In the other patient, RVAT_95_ was reduced by NS-HBP even when S-HBP did not result in any RVAT_95_ reduction, indicated failed bundle recruitment but resynchronization through multiple right ventricular wavefronts.

# **4. Supplementary Figures**

|  |
| --- |
| **Supplementary Figure S1: Activation Times (all patients)**  All six panels show data for all patients (n = 20). In the group of all patients, NS-HBP prolongs neither left nor right ventricular activation time compared to S-HBP and Intrinsic rhythm. **Top Left (A):** Change in LVAT_95_ from intrinsic rhythm to S-HBP (left) and intrinsic rhythm to NS-HBP (right). **Top Middle (B)**: RVAT_95_ from intrinsic rhythm to S-HBP (left) and intrinsic rhythm to NS-HBP (right). **Top Right (C)**: QRSd from intrinsic rhythm to S-HBP (left) and intrinsic rhythm to NS-HBP (right). **Bottom left (D)**: LVAT_95_ in intrinsic rhythm (left), NS-HBP (middle) and S-HBP (right). **Bottom middle (E)**: RVAT_95_ in intrinsic rhythm (left), NS-HBP (middle) and S-HBP (right). **Bottom right (F)**: QRSd in intrinsic rhythm (left), NS-HBP (middle) and S-HBP (right).  S-HBP – selective His bundle pacing; NS-HBP – non-selective His bundle pacing; LVAT_95_ – Left Ventricular Activation Time of 95% of activations. RVAT_95_ = Right Ventricular Activation Time of 95% of activations. |

| 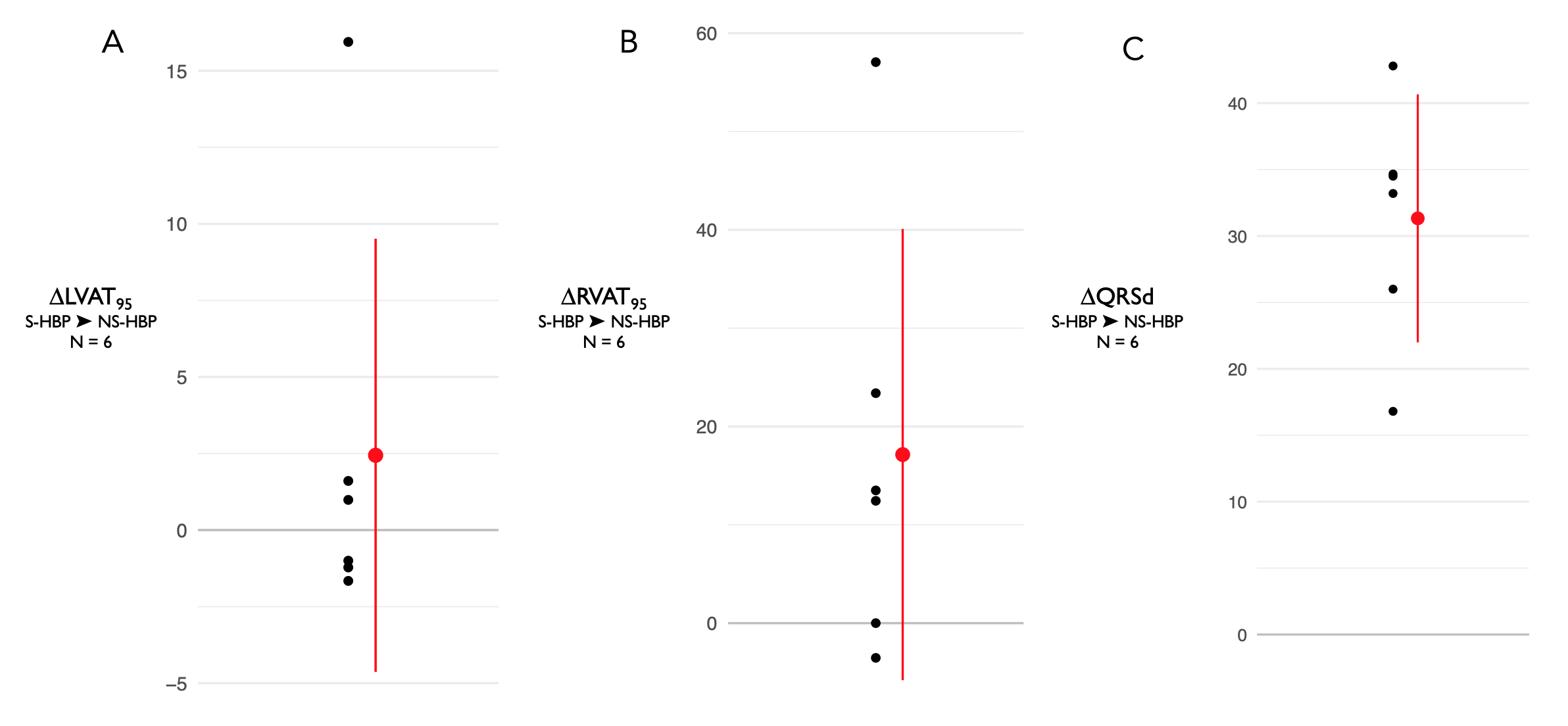 |
| --- |
| **Supplementary Figure S2: Change in Activation Times from Selective HBP to Non-selective HBP (patients with narrow intrinsic QRS)**  Change from S-HBP to NS-HBP for LVAT_95_ (left), RVAT_95_ (middle), QRSd (right) is shown for patients with an intrinsically narrow QRS (n=6).  S-HBP – selective His bundle pacing; NS-HBP – non-selective His bundle pacing; LVAT_95_ – Left Ventricular Activation Time of 95% of activations. RVAT_95_ = Right Ventricular Activation Time of 95% of activations. |

|  |
| --- |
| **Supplementary Figure S3 – Example ECGs and Example of Sub-Hisian Block**  Left: An example of transition between S-HBP and NS-HBP. Right: An example of Sub-Hisian complete heart block with HV dissociation. NS-HBP will allow continuous ventricular pacing if distal sub-Hisian conduction block occurs while S-HBP will not.  S-HBP – selective His bundle pacing; NS-HBP – non-selective His bundle pacing |
